# A Case of Life-Threatening Postpartum Necrotizing Pancreatitis Requiring Gastrocystostomy With Serial Pancreatic Necrosectomies

**DOI:** 10.7759/cureus.57779

**Published:** 2024-04-07

**Authors:** Adam Mangold, Emma Frost, Ted Raddell, Alexander Schoifet, Ziqian Wang

**Affiliations:** 1 Medicine, Cooper Medical School of Rowan University, Camden, USA; 2 Neurology, Cooper Neurological Institute, Cooper University Hospital, Camden, USA; 3 Internal Medicine, Cooper University Hospital, Camden, USA; 4 Hospital Medicine, Cooper University Health Care, Camden, USA

**Keywords:** anasarca, source control, pancreatic necrosectomy, gastrocystostomy, acute necrotizing pancreatitis

## Abstract

We present a life-threatening case of postpartum acute necrotizing pancreatitis. The patient is a 37-year-old female with no past medical history who delivered a healthy baby boy via cesarean section. Twenty days later, she presented to the emergency department with acute onset of nausea, non-bloody vomiting, abdominal bloating, and epigastric pain radiating to the back. Less than 24 hours later, she progressed into septic shock despite aggressive resuscitation, requiring vasopressor support in the ICU. Initial CT imaging showed multiple patchy hypodensities throughout the pancreas consistent with severe necrotizing pancreatitis. Her hospitalization was further complicated by difficulty obtaining source control of her infection, *Clostridium difficile*, and nutritional deficiencies that resulted in gross anasarca. She was discharged from the hospital on day 59 after undergoing multiple percutaneous drain placements, IV antibiotics, and endoscopic gastrocystostomy with four pancreatic necrosectomies. Since discharge, the patient has required readmission twice for complications from her pancreatitis.

## Introduction

Acute idiopathic pancreatitis, which can become necrotizing, has had a rising incidence in the United States in recent years [1] but has remained somewhat rare among the pregnant population. The incidence is approximately one in 1,000 to one in 10,000 with only 30% of those occurring during the postpartum period [2]. Acute pancreatitis in the postpartum period is most commonly due to gallstone pancreatitis, the development of biliary sludge, or a combination of both, especially in women of advanced maternal age [3]. Pregnant patients are predisposed to gallstones secondary to cholestasis of pregnancy for which the mechanism is not entirely understood, however genetic, hormonal, and environmental factors are all proposed to play a role [4]. A clinical diagnosis of acute pancreatitis can be made with two of the following three: characteristic abdominal pain (sharp, stabbing, radiating to the back), a marked elevation in pancreatic enzymes (lipase, amylase), and abdominal imaging consistent with pancreatic inflammation. Approximately 20% of patients with acute pancreatitis are complicated by the necrotizing form of the disease, which carries a mortality risk as high as 20-30% [5,6]. Rates are likely higher in postpartum patients, although the true rate remains unknown and there is little speculation in the literature as to a definite cause. Here, we present a life-threatening case of postpartum acute necrotizing pancreatitis complicated by septic shock ultimately requiring advanced endoscopic intervention.

## Case presentation

**Figure 1:**
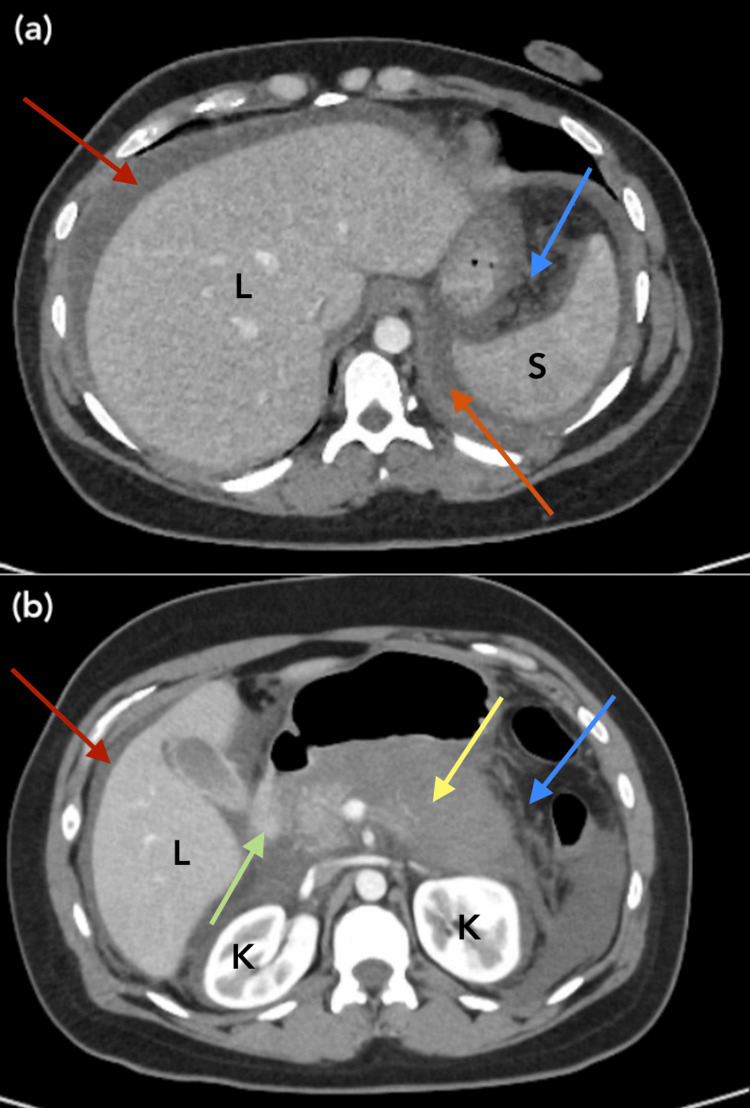
Representative images (a, b) of CTAP w/IV contrast from CUH day two. Note the poorly defined nature of the pancreatic body (yellow arrow) due to the extensive, damaging necrotizing process L: Liver; S: Spleen; K: Kidney Red arrow: Perihepatic ascites Orange arrow: Pronounced fluid collection tracking near the spleen, consistent with peripancreatic edema Yellow arrow: Poorly-defined, markedly hypoenhancing pancreatic body and tail, consistent with acute necrosis Green arrow: A more normal-appearing/properly enhancing area of pancreatic head tissue, in stark contrast to the hypoenhancement of the remainder of the pancreas Blue arrow: Peripancreatic edema and fat stranding secondary to necrosis Abbreviations: CTAP: Computed tomography of abdomen/pelvis; IV: Intravenous; CUH: Cooper University Hospital

A 37-year-old female with no significant past medical history gave birth to a healthy baby boy via cesarean section with a bilateral tubal ligation. She has no family history of pancreatitis and denies a personal history of alcohol or recreational substance use. This was her third child, with all three pregnancies ending in delivery via cesarean section. Twenty days later (outside hospital (OSH) day one), she presented to the emergency room with acute onset of nausea, non-bloody non-bilious vomiting, abdominal bloating, and sharp, stabbing epigastric pain that radiated to the flank/back and worsened with eating. Abdominal CT on OSH day three revealed hepatic steatosis, minimal intrahepatic duct dilation, a common bile duct measuring 6 mm, moderate abdominal ascites, and mesenteric ischemia. At that time, she was found to have a splenic vein and superior mesenteric vein thrombus and was started on a therapeutic heparin infusion. Imaging findings in conjunction with characteristic abdominal pain, elevated lipase, and leukocytosis led to a diagnosis of acute necrotizing pancreatitis. The patient became hypertensive at OSH, which sparked concern for preeclampsia in the setting of a recent delivery. She was then transferred to Cooper University Hospital (CUH) under the maternal-fetal medicine service for escalation of care. Upon admission to CUH, her labs remained unchanged and imaging demonstrated a worsening of her acute necrotizing pancreatitis (Figure 1).

She was treated aggressively with IV fluid resuscitation, antiemetics, and IV magnesium for seizure prophylaxis. On CUH day two, she was upgraded to the intensive care unit (ICU) due to persistent hypotension and lactic acidosis despite fluid resuscitation. While in the ICU, therapeutic anticoagulation was discontinued due to increased bleeding risk.

On CUH day four, she went into septic shock requiring vasopressor support via Levophed at a maximum dose of 20 mcg/min for two days before being weaned off to normotension. Meropenem was initiated as blood cultures returned positive for *Escherichia coli.* Meropenem was selected as carbapenem antibiotics have excellent deep tissue penetration and are preferred due to their coverage of gram-negative and anaerobic bacteria, especially in complicated, potentially multi-drug resistant, intraabdominal infections. She was restarted on heparin for her known mesenteric thrombus with mesenteric ischemia despite elevated bleeding risk in the setting of acute necrotizing pancreatitis. On CUH day nine, the patient was hemodynamically stable and downgraded to a general medicine floor, however, her leukocytosis persisted at 32,000/μL. Further workup showed a normocytic anemia with a hemoglobin of 8.5 g/dL and a platelet count of 286,000/μL. Her electrolytes were unremarkable, and her kidney function was preserved with a creatinine of 0.47 and adequate urine output. Her liver function tests including aspartate transaminase, alanine aminotransferase, alkaline phosphatase, and bilirubin were within normal limits. Her hospital course was further complicated by watery diarrhea secondary to *Clostridium difficile*, for which she was treated with fidaxomicin. Despite broad-spectrum antibiotic coverage for acute necrotizing pancreatitis and concurrent *C. diff* treatment, the patient continued to have febrile episodes and worsening leukocytosis. Repeat abdominal CT imaging on CUH day nine, unfortunately, revealed a new, ill-defined peripancreatic fluid collection with partial rim enhancement, consistent with large, walled-off necrosis, multiple abdominal fluid collections, worsening ascites, and reactive colitis. At this point, it became apparent that despite valiant efforts to curb the patient’s intraabdominal infection with careful tailoring of antibiotics, treatment of *C. diff*, diagnostic and therapeutic paracenteses for ascites, and thoracentesis for pleural effusion, inadequate source control was the main factor behind the patient’s persistent illness.

**Figure 2:**
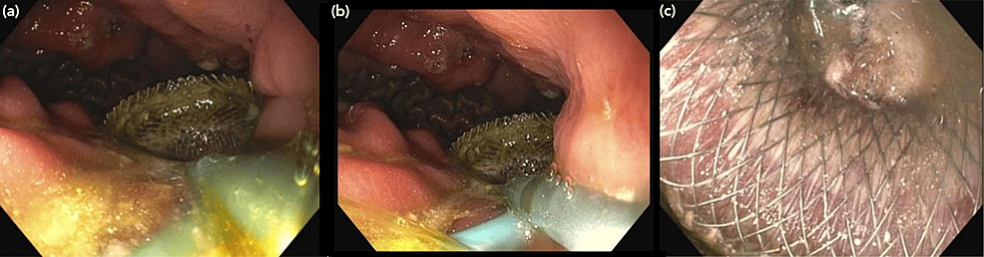
EGD images (a, b, c) taken on CUH day 45 during placement of the gastrocystostomy stent showing the stent being passed via endoscope with one end in the antrum of the stomach and the other in the pancreatic collection EGD: Esophagogastroduodenoscopy; CUH: Cooper University Hospital

**Figure 3:**
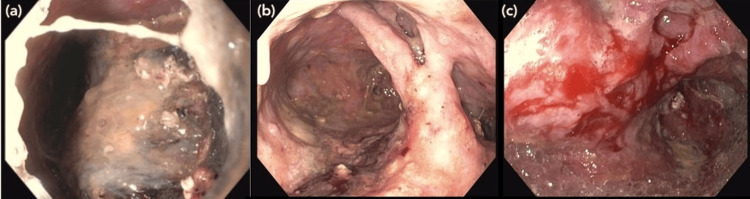
EGD images showing the patient’s pancreatic necrosis and wall of the stomach and duodenum before (a, b) and after (c) the first round of pancreatic necrosectomy on CUH day 45 EGD: Esophagogastroduodenoscopy; CUH: Cooper University Hospital

After a multidisciplinary discussion, the patient received further procedural interventions with interventional radiology (IR) and gastroenterology (GI). Anterior abdominal and retroperitoneal percutaneous drains were placed by IR with multiple subsequent drain exchanges. A gastrocystostomy AXIOS^TM^ stent (Boston Scientific, Marlborough, Massachusetts) (Figure 2) was placed by GI, followed by four pancreatic necrosectomies occurring on CUH days 45, 48, 56, and 58 (Figure 3).

It was a constant challenge to balance the pursuit of infectious source control with procedural efforts that required fasting, as the patient’s already poor nutritional status exacerbated her anasarca. Her hospital course ultimately lasted 59 days before she became stable enough for discharge to a rehabilitation facility with two percutaneous drains and a peripherally inserted central catheter in place for outpatient IV antibiotics. After one short day at the rehabilitation facility, the patient required readmission to the hospital after a dislodgment of her percutaneous drain. Her readmission required an additional 12 days, during which her retroperitoneal drain was exchanged, her anterior abdominal drain was removed, and an additional round of necrosectomy was completed prior to discharge. Her overall time course, including her readmission, totaled 73 days. Thankfully, her recovery progressed smoothly at home, and she has not required any additional hospitalizations for complications related to her necrotizing pancreatitis.

## Discussion

In acute pancreatitis, intrapancreatic activation of pancreatic enzymes causes direct cellular and tissue injury, with proteolytic and lipolytic enzymatic activities leading to parenchymal necrosis [5,6]. This case is an unfortunate example of how volatile acute necrotizing pancreatitis can be and raises several questions. Pertaining to pre-hospitalization care, could her life-threatening infection have been prevented if she had not attributed these symptoms to postpartum recovery and therefore sought care sooner? How can providers screen and remain vigilant for cholestasis and choledocholithiasis, which could potentially lead to pancreatitis, in the postpartum setting? Can we, as providers, do a better job educating patients on expected postpartum symptoms and how to recognize warning signs that require urgent attention? It is currently unclear if being acutely postpartum contributed to the severity of the infection or put the patient at higher risk for gallstone pancreatitis in the first place. More studies are needed to look at the association of necrotizing pancreatitis with postpartum hormonal changes and body composition to determine if there is any association.

Pertaining to the hospitalization itself, how can source control be achieved in a more effective and efficient manner for intraabdominal fluid collections that cannot be adequately drained via percutaneous drain placement? Despite multiple drain placements with IR, this patient remained septic due to a persistent retrogastric collection not amenable to percutaneous drainage, rendering source control nearly impossible.

The traditional management of acute necrotizing pancreatitis utilized invasive surgical debridement; however, minimally invasive techniques recently became standard of care due to their decreased rate of complications and lower mortality [7,8]. A “step-up” approach is now used in treatment, beginning with antibiotic coverage and progressing to minimally-invasive drainage, generally performed either endoscopically or laparoscopically, and necrosectomy as necessary [9]. In this case, a lumen-apposing self-expandable metallic stent and delivery system were implemented via endoscopic ultrasound guidance to facilitate the creation of a gastrocystostomy between the walled-off, difficult-to-access necrosis, and the patient’s stomach. By deploying this stent, the presumed source of infection drained naturally through the stomach lumen as opposed to via the placement of an additional external drain, which would carry its own inherent risk and be unlikely to succeed given the inaccessibility of the fluid pocket. Previously, plastic stents were found to migrate easily and self-occlude, requiring frequent replacement and repair. However, a metallic stent, including the stent used in this case, has multiple advantages, including its larger diameter, apposition, and reduced risk of stent migration [10]. These stents have been shown to be relatively safe, with rare adverse events reported during their placement or removal, especially when placed with the aid of endoscopic ultrasound [10]. The use of metallic stents to drain pancreatic fluid collections is becoming better described in the literature, with multiple case reports from the United States, Europe, and Japan providing well-tolerated and effective examples of these stents in cases of complicated acute necrotizing pancreatitis [10].

Once the stent was installed, endoscopic pancreatic necrosectomy occurred episodically, as needed, through the stent without significant worry of stent failure, migration, or dislodgement. The literature shows the technical success of these specific metallic stents at 96% without significant mortality or side effects and clinical success at 70-80%, making their use a safer alternative to more invasive procedures [10-12]. Had this patient not been at an institution where advanced endoscopy was readily available, it is possible that her overall outcome would have been significantly worse. Advancements in this field have begun to improve morbidity and mortality at a rapid rate.

It was not lost on the provider team how emotionally vulnerable and torturous it was for the patient, a new mother, to be acutely ill and away from her newborn throughout this extended hospitalization. Novel, creative solutions are needed to address emotional difficulties in navigating motherhood during prolonged hospitalizations, as in this patient’s case.

## Conclusions

There are few reports of endoscopic gastrocystostomy being used in the treatment of necrotizing pancreatitis, with a particularly novel application, in this case, addressing the retrogastric fluid collection that rendered source control nearly impossible. There are even fewer cases reported in patients who are acutely postpartum. Further investigations are needed to assess the efficacy and safety profile of this technique in the context of necrotizing pancreatitis complicated by inadequate source control, especially in postpartum women. We hope that this case report will spark conversation and research efforts in addressing these questions and challenges and ultimately lead to better patient outcomes.
